# Glial cell type-specific changes in spinal dipeptidyl peptidase 4 expression and effects of its inhibitors in inflammatory and neuropatic pain

**DOI:** 10.1038/s41598-018-21799-8

**Published:** 2018-02-22

**Authors:** Kornél Király, Márk Kozsurek, Erika Lukácsi, Benjamin Barta, Alán Alpár, Tamás Balázsa, Csaba Fekete, Judit Szabon, Zsuzsanna Helyes, Kata Bölcskei, Valéria Tékus, Zsuzsanna E. Tóth, Károly Pap, Gábor Gerber, Zita Puskár

**Affiliations:** 1 Department of Pharmacology and Pharmacotherapy, Semmelweis University, H-1089 Budapest, Hungary; 2 Department of Anatomy, Histology and Embryology, Semmelweis University, H-1094 Budapest, Hungary; 3 “Lendület” Laboratory of Integrative Neurobiology, Institute of Experimental Medicine of the Hungarian Academy of Sciences, H-1083 Budapest, Hungary; 4 Department of Pharmacology and Pharmacotherapy, Medical School & Szentágothai Research Centre, University of Pécs, H-7624 Pécs, Hungary; 5 MTA-PTE NAP B Chronic Pain Research Group, University of Pécs, H-7624 Pécs, Hungary; 6 Department of Traumatology, Semmelweis University, H-1113 Budapest, Hungary & Department of Orthopaedics and Traumatology, Uzsoki Hospital, H-1145 Budapest, Hungary

## Abstract

Altered pain sensations such as hyperalgesia and allodynia are characteristic features of various pain states, and remain difficult to treat. We have shown previously that spinal application of dipeptidyl peptidase 4 (DPP4) inhibitors induces strong antihyperalgesic effect during inflammatory pain. In this study we observed low level of DPP4 mRNA in the rat spinal dorsal horn in physiological conditions, which did not change significantly either in carrageenan-induced inflammatory or partial nerve ligation-generated neuropathic states. In naïve animals, microglia and astrocytes expressed DPP4 protein with one and two orders of magnitude higher than neurons, respectively. DPP4 significantly increased in astrocytes during inflammation and in microglia in neuropathy. Intrathecal application of two DPP4 inhibitors tripeptide isoleucin-prolin-isoleucin (IPI) and the antidiabetic drug vildagliptin resulted in robust opioid-dependent antihyperalgesic effect during inflammation, and milder but significant opioid-independent antihyperalgesic action in the neuropathic model. The opioid-mediated antihyperalgesic effect of IPI was exclusively related to mu-opioid receptors, while vildagliptin affected mainly delta-receptor activity, although mu- and kappa-receptors were also involved. None of the inhibitors influenced allodynia. Our results suggest pathology and glia-type specific changes of DPP4 activity in the spinal cord, which contribute to the development and maintenance of hyperalgesia and interact with endogenous opioid systems.

## Introduction

DPP4 is a type II integral transmembrane glycoprotein expressed on many cell types, but appears also in soluble form in body fluids including cerebrospinal fluid^1^. As a serine protease, DPP4 cleaves dipeptides from oligopeptides and proteins containing proline/alanine in the penultimate position. DPP4 processes neuropeptides, hormones, cytokines and chemokines leading to their biological activation or inactivation. Potential substrates include incretins (glucagon-like peptide-1 and -2, and glucose-dependent insulinotropic polypeptide), bradykinin, Substance P (SP), neuropeptide Y (NPY), vasoactive intestinal polypeptide (VIP) and tumour necrosis factor (TNF-α)^2–4^. In addition to the enzymatic activity, DPP4 has binding sites for adenosine deaminase (ADA)^5^ and different extracellular matrix proteins like collagen and fibronectin^6^. DPP4 is also known as cell surface antigen CD26 on T-lymphocytes^7,8^ and as a receptor for Coronaviruses^9^.

Incretins are the most familiar substrates of DPP4 since these hormones are major regulators of postprandial insulin secretion. Inhibition of DPP4 increases the incretin levels and prolongs the postprandial insulin action. Therefore DPP4 has become a major target for the therapy of type II diabetes. Application of newly developed DPP4 inhibitors revealed several physiological and pathological processes such as lipid metabolism, myocardial, renal and liver functions, atherosclerosis and inflammation in which DPP4 is involved^10,11^.

Control of chronic pain associated with tissue injury, inflammation or ongoing diseases have made no progress for decades. Current analgesics are at best moderately effective and associated with intolerable side effects. Therefore, development of novel therapeutic interventions for pain relief is one of the chief challenges for medical sciences. It is well established that altered pain sensations such as hyperalgesia (an increased response to noxious stimuli), allodynia (painful response to normally innocuous stimuli) and spontaneous pain are characteristic features of various pain states^12^. Previously we have demonstrated dramatic reduction of mechanical hyperalgesia following spinal application of DPP4 inhibitors (IPI and vildagliptin) in subacute inflammation and this action was naloxone reversible suggesting an opioid receptor-mediated effect. None of the inhibitors changed the nociceptive threshold in acute nociceptive tail-flick test^13^. Analgesic and anti-inflammatory effects of DPP4 inhibitors were also showed in chronic inflammatory models in mice^14^.

Machinery of the endogenous opioid system has been intensely investigated and clarified in recent decades. Although inducing/regulating the endogenous opioid machinery would provide a powerful tool to control pain propagation, this possibility has remained largely unexploited. Here, we identify DPP4 in the spinal dorsal horn, show that its expression changes during pathological conditions, and demonstrate that it shapes opioid signalling in a receptor- and treatment-specific manner. Although synaptic DPP4 may have a key role in neuronal mechanisms of pain propagation, we identify glial cells as inducible DPP4-batteries, in this way playing a role in hyperalgesia and opioid signalling.

## Results

### DPP4 transcripts in the rat spinal dorsal horn in physiological, inflammatory and neuropathic states

**Figure 1:**
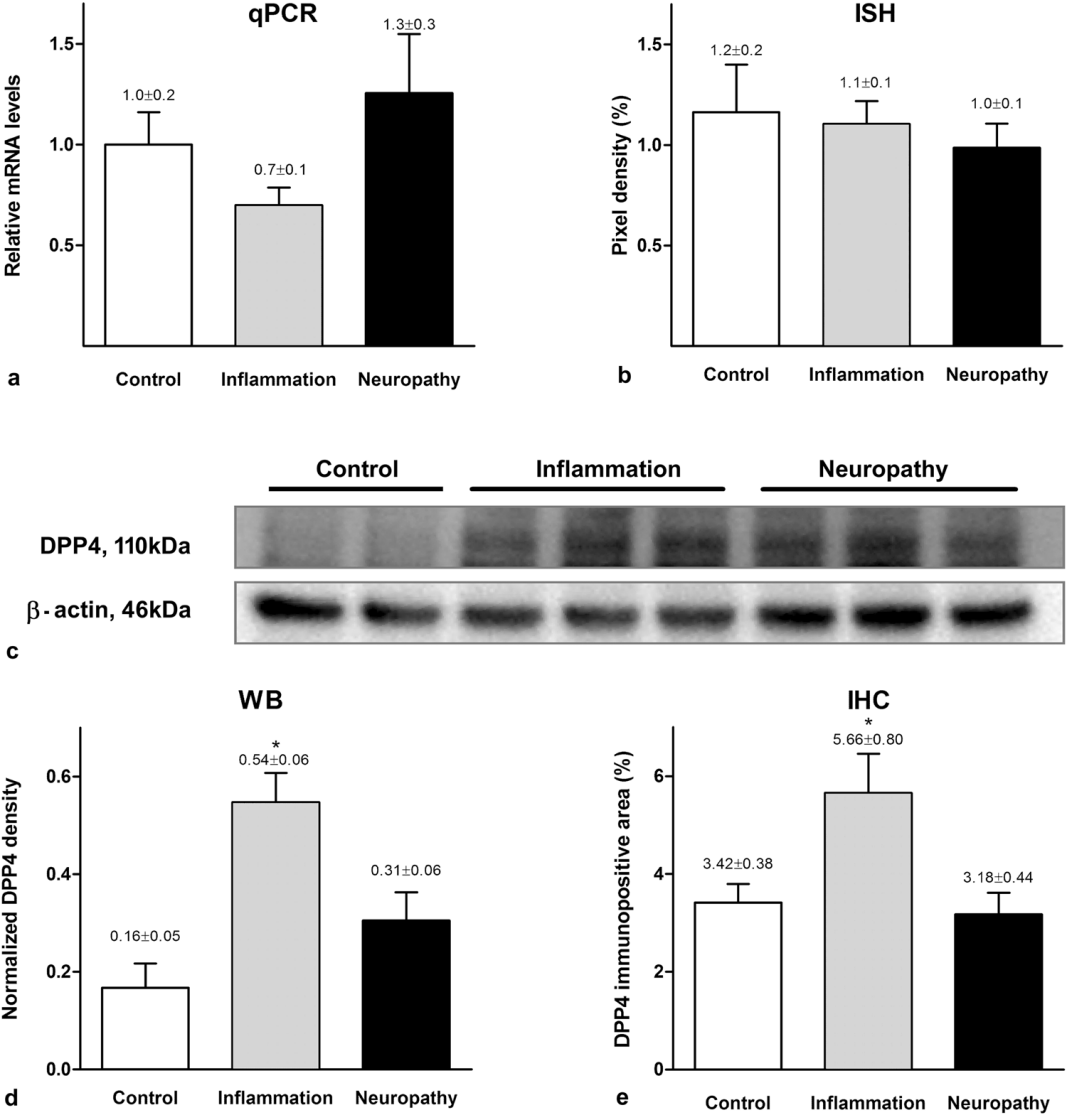
DPP4 mRNA and protein expression in the dorsal horn of control, carrageenan treated and neuropathic rats. DPP4 mRNA expression in the dorsal horn of the spinal cord assessed by qPCR (**a**) and *in situ* hybridization (**b**) did not show significant difference among the three experimental groups (mean ± SEM, n = 6–9, one-way ANOVA, P = 0.30 and P = 0.21 for qPCR and ISH, respectively). In Western-blot experiments goat DPP4 antibody labelled lane at 110 kDa in spinal dorsal horn lysates taken from naive, inflamed and neuropathic animals (**c**). The full gel is shown in Supplementary Figure S1. Significantly increased DPP4 protein levels were detected in carrageenan-induced inflammation measured both by Western-blotting (**d**) and quantitative immunohistochemistry (**e**). (Values are given as mean ± SEM, n = 7–10, one-way ANOVA followed by Holm-Sidac *post hoc* test: P = 0.023 for Western blot experiments and one-way ANOVA with Student-Neuman-Keuls *post hoc* test: P = 0.016 for densitometry).

Taqman qPCR detected DPP4 mRNA in the dorsal horn of L5 spinal segments taken from control, inflamed and neuropathic rats. Neither carrageenan treatment nor neuropathic condition caused significant alteration in the DPP4 mRNA levels (relative quantities in control, carrageenan-induced inflammation and neuropathic groups: 1.0 ± 0.2 *vs*. 0.7 ± 0.1 *vs*. 1.3 ± 0.3, one-way ANOVA P = 0.301; Fig. 1a).

DPP4 mRNA showed a low expression by *in situ* hybridization in the spinal dorsal horn of L4-L6 segments. While the grain density observed in sections hybridized using the sense probe was equal to the background, a significant signal was detected with the antisense probe (antisense: n = 27, median = 110.4, 25% at 80.7 and 75% at 135.5; sense: n = 19, median = 0.0, 25% at 0.0, 75% at 21.7; Mann-Whitney Rank Sum Test P < 0,001). DPP4 mRNA was distributed evenly within the dorsal horn, and no significant differences among the experimental groups were detected (control: n = 9, 1.2 ± 0.2; inflamed: n = 9, 1.1 ± 0.1; neuropathic: n = 9, 1.0 ± 0.1; one-way ANOVA, P = 0.21; Fig. 1b).

The data obtained from both qPCR and *in situ* hybridisation studies clearly demonstrated that DPP4 mRNA exists in the spinal dorsal horn and its quantity does not change either in inflammatory or neuropathic pain states.

### DPP4 protein expression in physiological condition and its changes during inflammation and neuropathy in the spinal dorsal horn

Western blot banding pattern of DPP4 originated from rat spinal dorsal horn appeared very similar to that one which was taken from human white (pre)adipocytes using an antibody different from the one used in the current experiments^15^. Western-blot analysis in this study demonstrated an elevated protein level in the inflamed spinal cord being significantly different from those found in naïve and neuropathic spinal cords (control: 0.16 ± 0.05; inflamed: 0.54 ± 0.06; neuropathic: 0.31 ± 0.06; one-way ANOVA, Holm-Sidak method, P = 0.023; Fig. 1c-d).

DPP4 immunoreactivity in the spinal cord appeared in naïve, inflamed and also in neuropathic animals. Densitometry of the DPP4 immunolabelling showed a significant increase in the medial two third of the dorsal horn (corresponding to the hind paw) during inflammation compared to naïve and neuropathic conditions (control: n = 7, 3.42 ± 0.38; inflamed: n = 10, 5.66 ± 0.80; neuropathic: n = 8, 3.18 ± 0.44; one-way ANOVA P = 0,016; Fig. 1e).

In contrast to DPP4 mRNA, the overall amount of DPP4 protein detected by Western-blot and immunohistochemistry significantly increased in the spinal dorsal horn during inflammation and did not change in neuropathy compared to the physiological state.

### DPP4 immunoreactivity and its changes in individual cell types during pathology

**Figure 2:**
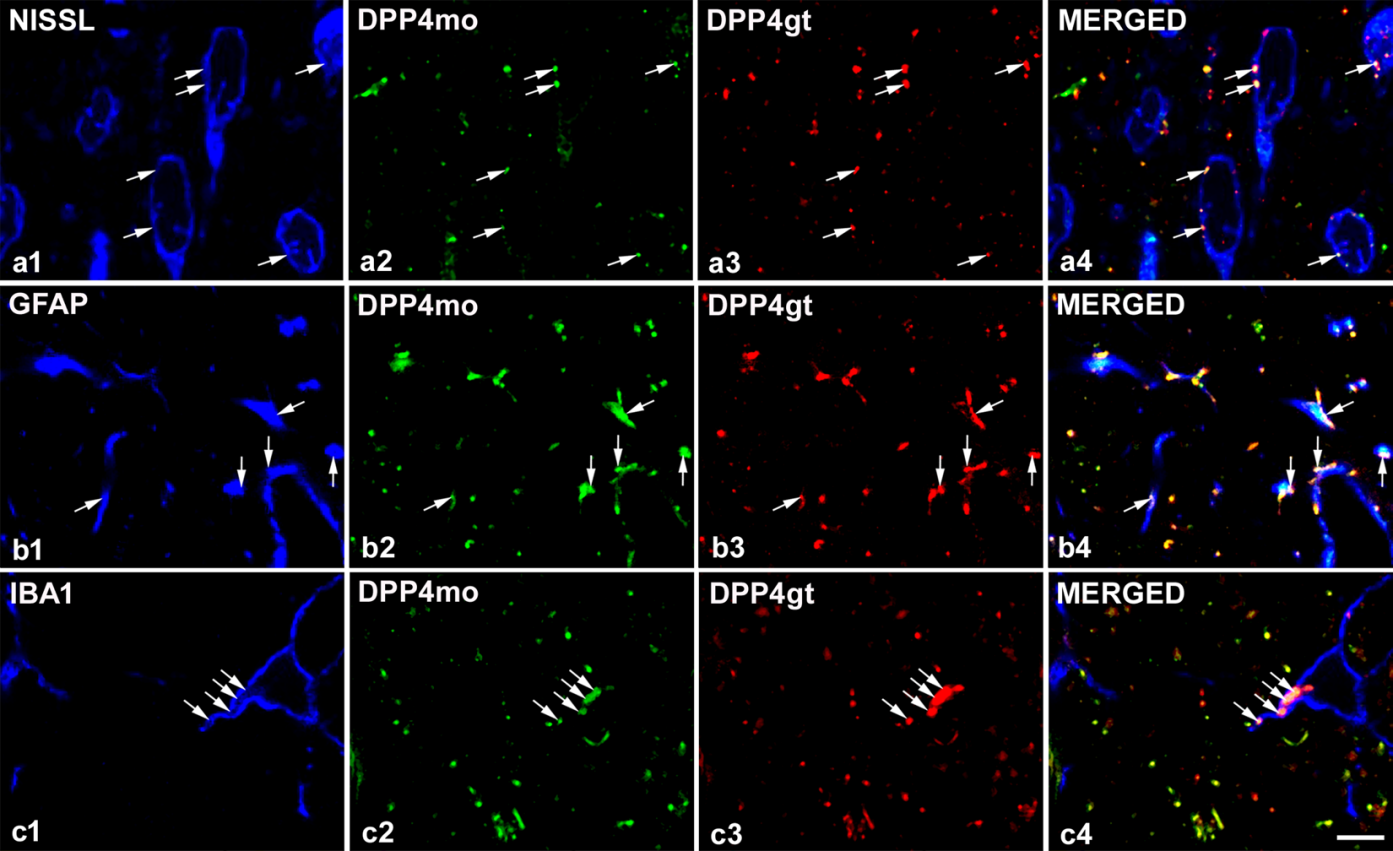
DPP4 immunoreactivity of different cell types in the spinal dorsal horn. DPP4-immunoreactive puncta appeared in Nissl stained neurons (row a), GFAP labelled astrocytes (row b) and IBA1 positive microglial cells (row c). Co-staining with mouse monoclonal antibody against the full length rat CD26 protein (DPP4mo, column 2) and polyclonal goat DPP4 antibody that was raised against the synthetic peptide C-PPHFDKSKKYP representing the internal region of DPP4 (DPP4gt, column 3) labelled the same puncta in all three cell types demonstrating the specificity of the two antibodies (arrows). All the images are single optical sections. Scalebar: 5 μm.

**Figure 3:**
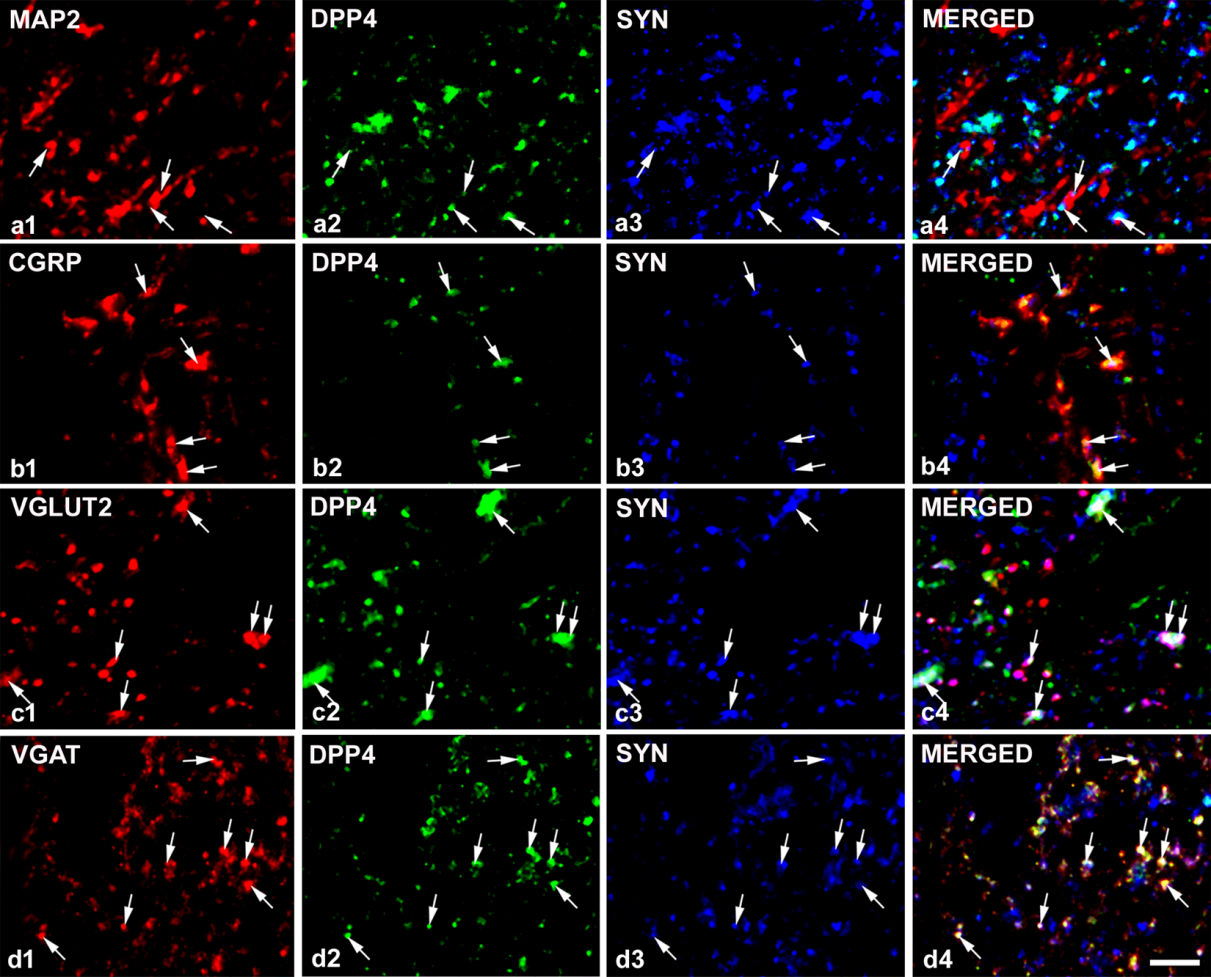
DPP4 immunoreactivity in various types of axon terminals. DPP4 immunolabelling on MAP2-stained dendritic surfaces was associated with synaptophysin (SYN) indicating that not the dendrites but the axon terminals express the receptor (row a). DPP4 is expressed by the majority of peptidergic (CGRP-containing) C primary afferent terminals (row b) and coexpression of DPP4 with SYN and VGLUT2 (row c) or VGAT (row d) suggests that DPP4 is present both in excitatory and inhibitory nerve endings. All the images are single optical sections. Scalebar: 5 μm.

Punctate immunostaining was detected in neuronal cell bodies (Fig. 2a) and also in axon terminals in naive animals. Puncta representing DPP4 were embedded in synaptophysin stained elements suggesting close relationship between synaptic and DPP4 activity in many cases **(**Fig. 3**)**. DPP4-immunopositive dots that appeared on the surface of MAP2 labelled dendrites were always associated with synaptophysin positivity **(**Fig. 3a**)** suggesting that dendrites did not express the enzyme but received synapses from DPP4-containing boutons. The enzyme appeared both in vesicular glutamate transporter 2 (VGLUT2) immunolabelled excitatory **(**Fig. 3c**)** and in vesicular GABA transporter (VGAT) positive inhibitory axon terminals **(**Fig. 3d**)**, as well as in CGRP stained primary afferent boutons **(**Fig. 3b**)**. DPP4 labelling also occurred in GFAP-positive astrocytes (Fig. 2b**)** and IBA1-stained microglia cells **(**Fig. 2c**)**. In naive rats, the density of the enzyme staining was the highest in astrocytes and differed significantly from that in other cell types. DPP4 density was also significantly higher in microglia than in neurons (astrocyte: n = 109, median = 18652.86, 25% at 8185.08, 75% at 31208.90; microglia: n = 83, median = 3196.484, 25% at 2338.702, 75% at 5189.73; neuron: n = 96, median = 343.95, 25% at 247.46, 75% at 420.69; Kruskal-Wallis one-way ANOVA on ranks, Dunn’s method at P < 0.001). During inflammation, DPP4 expression increased significantly in astocytes but not in microglia and neurons. Significant increase in the DPP4 immunoreactivity appeared only in microglia and significant decrease in neurons in the Seltzer model (astrocyte control: n = 109, median = 18652.86, 25% at 8185.08, 75% at 31208.90, inflammation: n = 112, median = 33379.28, 25% at 7651.47, 75% at 43072.64, Seltzer: n = 80, median = 15583.72, 25% at 8802.54, 75% at 22288.04, Kruskal-Wallis one-way ANOVA on ranks, Dunn’s method at P < 0.001; microglia control: n = 83, median = 3196.48, 25% at 2338.70, 75% at 5189.76, inflammation: n = 86, median = 2996,71, 25% at 2446.60, 75% at 4359.58, Seltzer: n = 67, median = 4926.80, 25% at 3312.32, 75% at 6493.72, Kruskal-Wallis one-way ANOVA on ranks, Dunn’s method P < 0.001; neuron control: n = 96, median = 343.95, 25% at 247.46, 75% at 420.69, inflamation: n = 64, median = 312.08, 25% at 203.53, 75% at 416.45, Seltzer: n = 64 median = 278.46, 25% at 213.09, 75% at 362.67, Kruskal-Wallis one-way ANOVA on ranks, Dunn’s method at P < 0.001, in all cases n means the number of the analysed optical sections of neuronal or glial elements). It should be noted that DPP4 immunolabelling existed not only in the membranes of the different cell types but also in their intracellular compartments.

**Figure 4:**
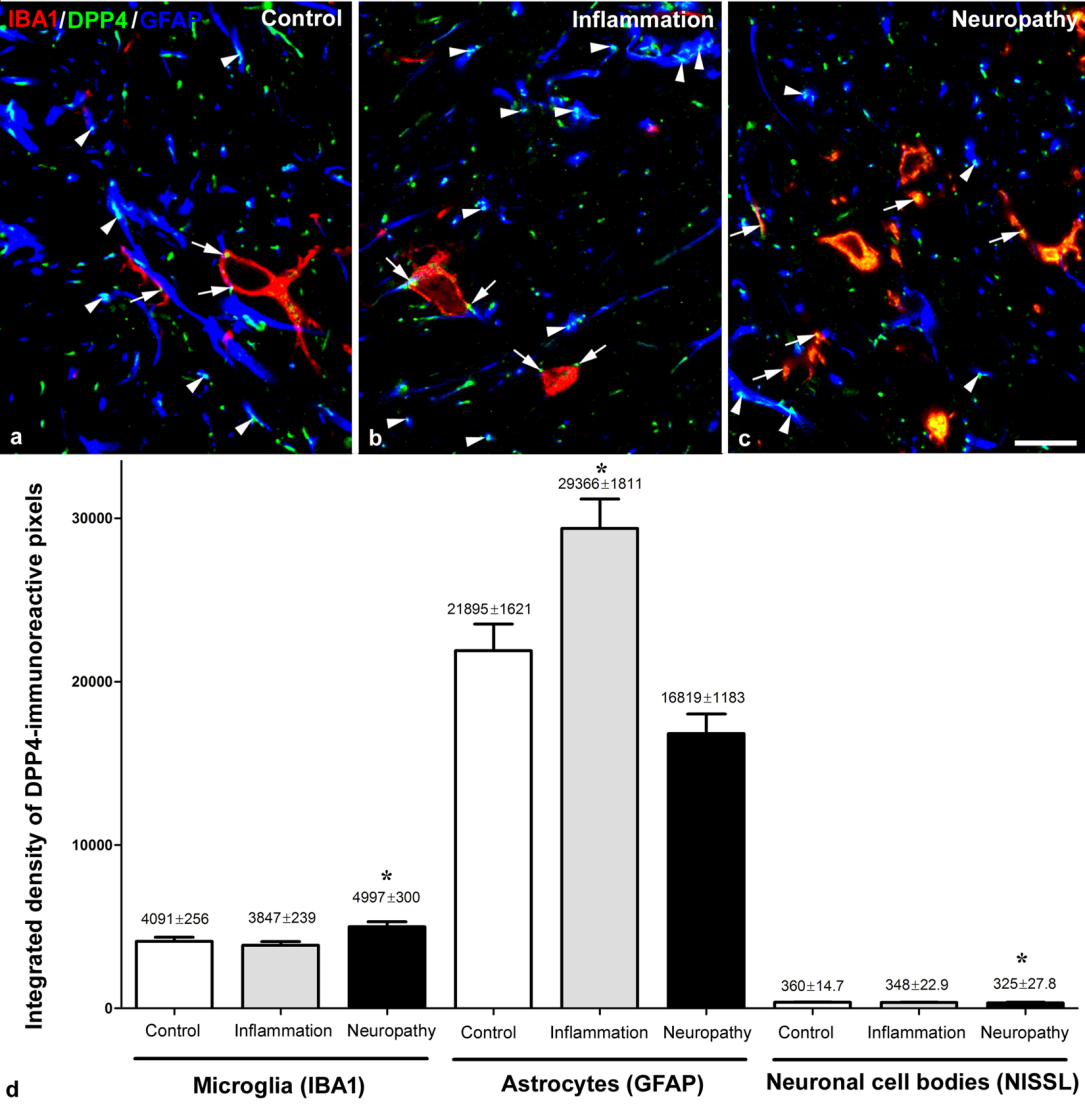
DPP4 immunoreactivity in glial cells in control, inflamed and neuropathic animals. Representative confocal images of the spinal dorsal horn obtained from control (**a**), inflamed (**b**) and neuropathic (**c**) animals demonstrate that DPP4 is expressed predominantly by glial cells (arrows: microglia, arrowheads: astrocytes). All the images are single optical sections. Scalebar: 10 μm. Integrated density values (**d**) demonstrate that the majority of DPP4-immunopositivity is related to GFAP-labelled astrocytes. A proportion of DPP4 labelling belongs to microgila and very few DPP4 is expressed by neuronal cell bodies. Inflammation significantly increased the DPP4 expression on astocytes but not on the other cell types. In contrast to inflammation DPP4 density was significantly higher on microglia and lower in neurons during neuropathy (Kruskal-Wallis One way ANOVA on ranks, Dunn’s Method, P < 0,001).

Taking the qualitative and quantitative data together **(**Table 1, Fig. 4**)**, DPP4 immunostaining was detected in neuronal cell bodies and also in excitatory and inhibitory axon terminals in naïve animals. Densitometric analysis showed that in naïve animals, microglia and astrocytes expressed DPP4 protein with one and two orders of magnitude higher than neurons, respectively. Furthermore, DPP4 expression significantly increased in astrocytes and did not change in other cell types during inflammation. In neuropathic conditions, DPP4 immunoreactivity significantly increased in microglia, decreased in neurons and remained unchanged in astrocytes.

**Table 1:** Changes of DPP4 expression of different cell types in pathological conditions.

Cell types	Inflammation	Neuropathy
Neurons	—	↓
Astrocytes	↑	—
Microglia	—	↑

A brief summary of how DPP4 expression changes in different cell types in different pathological conditions. Increases and decreases are indicated by upward and downward arrows, respectively.

### Opioid receptor types involved in the antihyperalgesic effect of DPP4 inhibitors in inflammation

**Figure 5:**
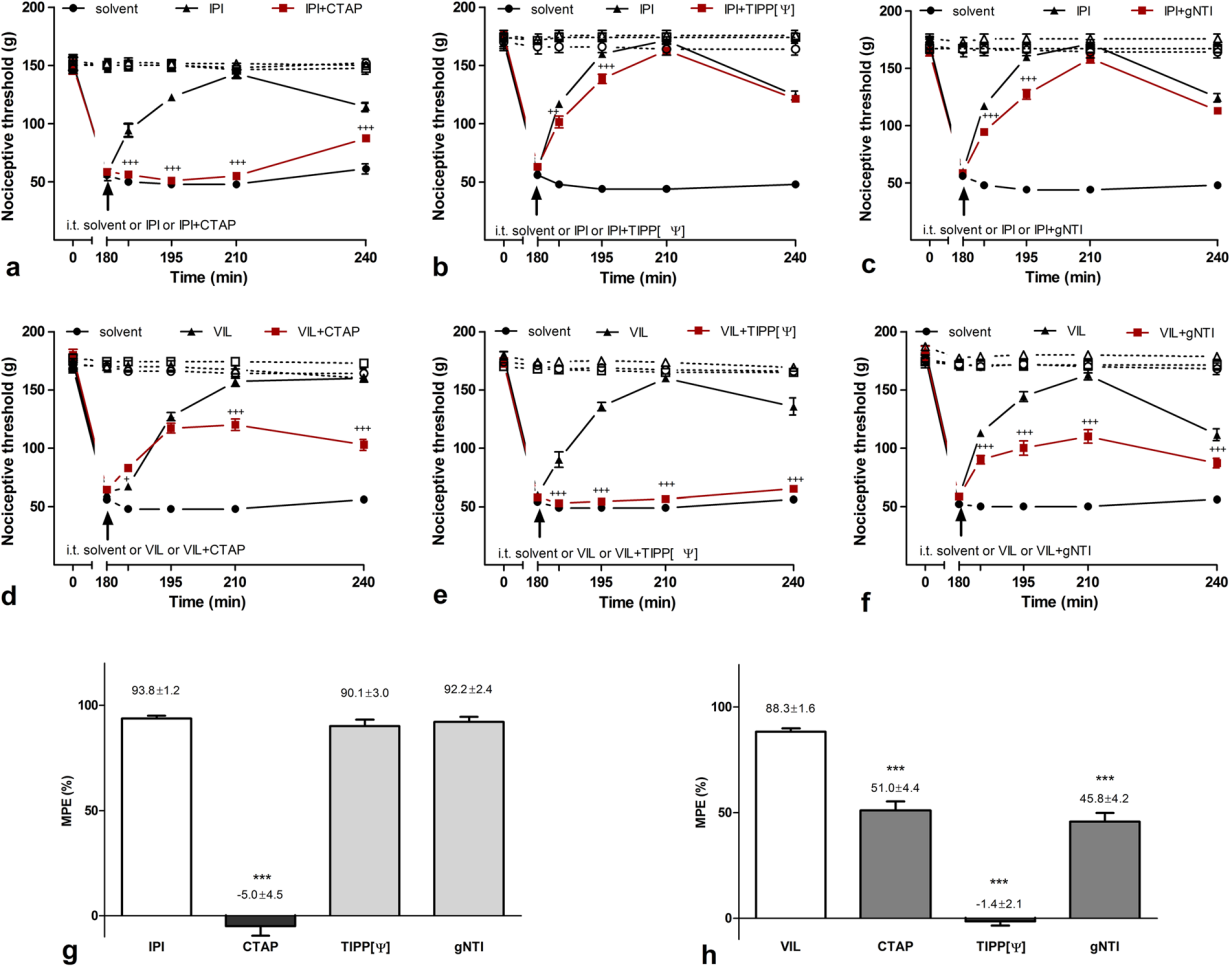
Antihyperalgesic effect of DPP4 inhibitor IPI and vildagliptin in carrageenan-induced inflammation. (**a**) Antihyperalgesic effect of IPI has been completely abolished by co-administartion of CTAP, but (**b**) neither TIPP[Ψ] nor (**c**) gNTI altered IPI-evoked antihyperalgesia. (**d**) Antihyperalgesic effect of vildagliptin was significantly reduced by CTAP and (**f**) gNTI, but was completely eliminated when (**e**) TIPP[Ψ] was co-injected. Inhibitory effects of opioid antagonists on IPI and vildagliptin related antihyperalgesia are summarized on bar graphs (**g**) and (**h**) constructed from data recorded 210 min after i.t. drug application. Comparisons were made with two-way ANOVA, Bonferoni *post hoc* test; ^+^p < 0.05; ^+++^p < 0.001 (**a**–**f**) and one-way ANOVA followed by Dunnett’s *post hoc* test. ***p < 0.001 (g and h). Asterisks always indicate significant differences between the time-matching points of *DPP4 inhibitor* and *DPP4 inhibitor* + *opioid antagonist* curves. Data on each curves and bars are given as mean and SEM.

To challenge the involved opioid receptor types, selective opioid receptor antagonists were applied spinally together with two different DPP4 inhibitors in carrageenan-induced subacute inflammation. I.t. application of 30 nmol/rat IPI and 3 nmol/rat vildagliptin eliminated 93.8 ± 1.2% and 88.3 ± 1.6% of mechanical hyperalgesia measured by the Randall-Selitto test in intraplantar carrageenan-induced inflammation. Co-administration of the mu-opioid receptor (MOR)-selective inhibitor CTAP reduced the antihyperalgesic effect of IPI to −5.0 ± 4.5%, while following co-application of kappa-receptor (KOR) antagonist gNTI and the delta-opioid receptor (DOR) antagonist TIPP[Ψ] the antihyperalgesic effect of IPI remained at 92.2 ± 2.4% and 90.1 ± 3.0%, respectively. Following co-administration of mu- and kappa-antagonists, antihyperalgesic effect of vildagliptin was 51.0 ± 4.4% and 45.8 ± 4.2%, respectively, while the delta-antagonist TIPP[Ψ] completely blocked the antihyperalgesic effect of vildagliptin by reducing its antihyperalgesic effect to −1.4 ± 2.1% **(**Fig. 5**)**.

These results show that both IPI and vildagliptin resulted in robust but distinct opioid-dependent antihyperalgesic effects during inflammation. The antihyperalgesic effect of IPI was fully blocked by the selective mu-opioid receptor antagonist and no decrease was observed by the selective kappa- and delta-antagonists. In case of vildagliptin, the antihyperalgesic effect of this inhibitor was completely blocked by selective delta-receptor antagonist while selective mu- and kappa-receptor antagonists also decreased the effect significantly.

### Effects of DPP4 inhibitors in neuropathy

**Figure 6:**
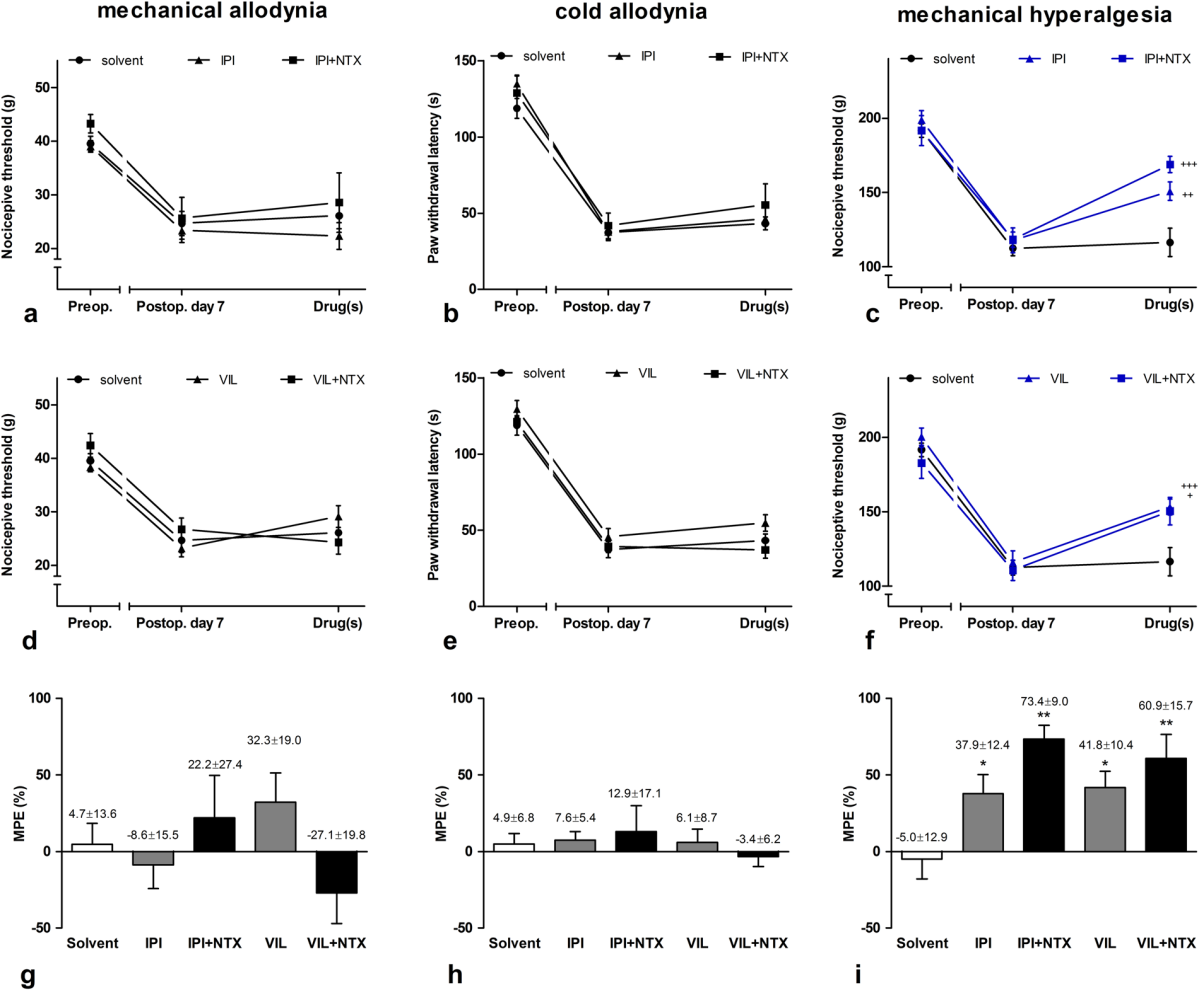
Antinociceptive effects of the DPP4 inhibitor IPI and vildagliptin in chronic neuropathic condition induced by partial sciatic nerve ligation. DPP4 inhibitors were ineffective in dynamic plantar aesthesiometer (**a** and **d**) and noxious cold sensitivity (**b** and **e**) tests. In Randall-Selitto test, both IPI and vildagliptin had antihyperalgesic effect which was not antagonized by NTX (**c** and **f**). Maximal possible effects (MPE%) of DPP4 inhibitors alone or in combination with subtype specific opioid antagonists is given on bar graphs (**g**–**i**). Comparisons were made with two-way ANOVA, Bonferoni *post hoc* test; ^+^p < 0.05; ^++^p < 0.01; ^+++^p < 0.001 (**a**–**f**) and one-way ANOVA followed by Dunnett’s *post hoc* test; *p < 0.1 **p < 0.01 (**g**–**i**). Data on each curves and bars are given as mean and SEM.

Different modalities of hyperalgesia and allodynia appear not only in inflammatory conditions but also in neuropathic pain states. None of the tested DPP4 inhibitors had significant effect on mechanical and cold allodynia, while both i.t. IPI and vildagliptin had a significant mechanical antihyperalgesic effect measured with the Randall-Selitto test one week after partial sciatic nerve ligation with MPE values of 37.9 ± 12.4% and 41.8 ± 10.4%, respectively. In contrast to inflammatory states, the nonselective opiate antagonist naltrexone (NTX) did not affect the antihyperalgesic action of the DPP4 inhibitors signifficantly in neuropathic conditions suggesting completely different actions of the enzyme on hyperalgesia in the two pain states **(**Fig. 6**)**.

## Discussion

A high level of DPP4 expression in the developing brain and spinal cord was described, which dramatically decreased in adults, but persisted in leptomeningeal cells and capillary endothelial cells of the choroid plexus^16–18^. DPP4 mRNA was detected in cortical areas in adult naïve animals and its level did not change after cerebral ischemia. In contrast, DPP4 immunoreactivity was not found in the same regions in physiological state but its expression appeared in microglia, neurons and astrocytes in different time points during cerebral ischaemia^19^. We are first to show that transcripts as well as the protein of DPP4 are detectable in the adult mammalian spinal dorsal horn. Similarly to the effects of ischemic injuries in the brain, our q-PCR and *in situ* hybridization analysis showed that DPP4 mRNA level was not influenced by inflammation or neuropathy in the spinal dorsal horn. The same treatments, however, caused marked changes of DPP4 protein level in this region. Inflamation caused a five-fold increase in DPP4 protein level suggesting a very potent posttranscriptional control of DPP4 expression during quickly developing inflammation and ischemia in neurons and glia. Our understanding of DPP4 molecular regulation is far from being complete. Studies on lymphocytes have demonstrated that while retinoic acid and interferon administration result in an increase of DPP4 transcription, IL12 upregulates only DPP4 translation and TNFα merely decreases the cell surface expression of DPP4^11,20^ suggesting a very complex mechanism of the regulation of DPP4 activity.

High resolution confocal laser scanning imaging revealed that neuronal DPP4 was typically confined to presynaptic, and also to somatic domains, with significantly decreased densities in neuropathy. In contrast, astrocytes were amply decorated with DPP4-immunoreactive profiles, with significantly increasing density in inflammatory but not in neuropathic states. Finally, a third-party involvement in spinal neuropathic mechanisms was likely reflected by the increase of DPP4 expression in microglia which showed an obvious protein expression level in control animals, too.

Detectable levels of the DPP4 protein in healthy spinal cord, in contrast to brain tissue, could be due to the quick on-demand regulatory role of the moonlight protein in nociceptive processes where SP and NPY offer ample and typical substrates for DPP4. The presynaptic location of DPP4 in neurons suggests a possible role for this protein in synaptic physiology. On the other hand, glial expression of DPP4 which is significantly upregulated in a pathology and cell type specific manner opens further aspects to detect alternative roles of this protein in different pain states.

Previously, we have demonstrated that DPP4 inhibitors do not change the nociceptive threshold in acute nociceptive condition^13^. In contrast to this, robust antihyperalgesic effects of different DPP4 inhibitors such as IPI, vildagliptin and sitagliptin in carrageenan-induced and complete Freund’s adjuvant (CFA)-provoked chronic inflammatory models have been reported previously^14,21^. In addition to the mechanical and heat hyperalgesia, mechanical and heat allodynia may also exist in the carrageenan-induced inflammatory model^22^. The two modalities of allodynia were not measured in this study since the effects of the non-noxious heat or mechanical stimuli cannot be detected very precisely because of the extreme large paw swelling. In the other hand, touch sensitivity measured by dynamic plantar aesthesiometry (DPA) in CFA-induced chronic inflammatory model showed no changes following intrathecal application of DPP4 inhibitors, although Randall-Selitto test demonstrated the antihyperalgesic effect (unpublished observation). These data suggest the spinal antihyperalgesic but not the antiallodynic effect of the DPP4 inhibitors in inflammatory conditions.

The antihyperalgesic action of IPI and vildagliptin appeared opioid-mediated in the carrageenan-induced inflammation since the nonselective opioid receptor antagonist naloxone/naltrexone reversed their effects^13,21^. In this study, we examined the opioid receptors involved by using selective antagonists against MOR, DOR, and KOR and measuring mechanical hyperalgesia in carrageenan-induced acute inflammation. Surprisingly, the antihyperalgesic effect of the IPI exclusively related to MOR, while vildagliptin affected mainly DOR but had also effect on MOR and KOR. It has been demonstrated previously that IPI does not activate MORs directly^23^. Both IPI and vildagliptin are inhibitors of the DPP4 but IPI with a penultimate proline is also a substrate of the enzyme and its competitive inhibition is a kinetic artefact^24^. Although both inhibitors target the active site of the enzyme, the extent of inhibition depends on the residual interaction between drug and active site residues. X-ray crystallography analysing the co-crystal structure of different inhibitors with DPP4 demonstrated that the inhibitors, but not the substrates could bind well beyond the S2 subsite to increase their inhibitory activity^25^. Taking all these together supports that different residual interactions of the two inhibitors can affect the DPP4 activity in different ways.

Endogenous opioids, especially enkephalins, dynorphins and endorphins, are released from spinal and supraspinal sites during acute inflammation, but are degraded very quickly by extremely high enzymatic activity^26^. A common feature of these opioids is that they can activate each opioid receptor with different potencies. Since both IPI and vildagliptin are very selective DPP4 inhibitors, it is very unlikely that it has inhibitory effect on opioid degrading enzymes. On the other hand, it has been demonstrated that glial cells express opioid receptors and can synthesize endogenous opioids^27–29^. These processes are at least partly regulated by inflammatory mediators including IL-1β^28^. The interaction between the two systems is mutual, since the endogeneous opioids also have effect on the production of inflammatory mediators released by glial cells^30^.

Glucagon-like peptide-1 (GLP1) receptor activation results in β-endorphin release from microglia and blocks inflammatory nociception and mechanical allodynia in the spinal nerve ligation model^27,31^. In this study, we have found an increase in the expression of DPP4 both in microglia and astrocytes that can facilitate the degradation of many peptides including GLP1. Systemic increase of GLP1 peptide has been detected during inflammation^32^ and IPI/vildagliptin blocking DPP4 activity may increase further the GLP1 level in the spinal cord and induce β-endorphin release from microglia.

In this study, we have found a substantial increase in the expression of DPP4 in astrocytes during inflammation. It has been also demonstrated that astrocytes can synthesize both proenkephalins and dynorphins^33,34^ that makes them good candidates for the source of these peptides. It has been shown that purinergic and toll-like receptor (TLR) activation results in dynorphin-A and -B releases from this glia type^34^ and proenkephalin release was also detected in cultured astrocytes^33^. However, the conditions in which astrocytes release proenkephalin *in vivo* have not been determined.

Recent *in vitro* studies in monocytes demonstrated that DPP4 inhibitors suppressed TLR4 mediated upregulation of proinflammatory cytokines including IL-1β, IL-6^35^, NLRP3 inflammasome which is a key molecule to process and release of IL-1β and IL-18^36^, and extracellular-regulated kinase (ERK) activation that has also critical role in expression of inflammatory cytokines^37^. These data suggest an important interaction between TLR4 and DPP4 activity which may exist also in glial cells and regulates the synthesis and release of inflammatory mediators and endogenous opioids.

Partial nerve injury is one of the main root causes of causalgiform pain disorders in human. Partial ligation of the sciatic nerve is a model of this pain state resulting in mechanical hyperalgesia together with mechanical and cold allodynia^38,39^. Although the altered pain sensation is a characteristic feature of both inflammatory and neuropathic pain states, very distinct anatomical and molecular mechanisms, as well as signalling pathways could be activated^12^. Therefore, DPP4 inhibitors were also tested in this model. Both IPI and vildagliptin produced a significant decrease in mechanical hyperalgesia, but this effect was not opioid-dependent. Comparing the extent of the antihyperalgesic effect of DPP4 inhibitors in inflammation and neuropathy may suggest that DPP4 inhibitors are more effective in inflammatory conditions then in neuropatic states. However, it must be emphasized that neuropathic pain and hyperalgesia are very difficult to treat and efficacy of DPP4 inhibitors is comparable to the adjuvant analgesic reference drugs used in the clinical practice in doses not causing sedative side-effect and motor incoordination^40^. Therefore, this moderate, but significant antihyperalgesic action should indeed be considered remarkable.

In addition to the mechanical hyperalgesia, mechanical and cold allodynia also appeared in this neuropathy. None of the DPP4 inhibitors affected either types of allodynia. Several models of different types of allodynia and hyperalgesia have been proposed with the common feature that the two phenomena based on different mechanisms^12,41–44^. The major issue is whether the specially designed behaviour tests for mechanical allodynia or mechanical hyperalgesia reflects these phenomena or not. In this study, the threshold-decrease determined with the Randall-Selitto test that applies a basically painful pressure stimulus on the rat hind paw is expected to show mechanical hyperalgesia. The touch stimulus applied by the DPA that utilizes a blunt-end metal needle is considered to be non-painful under intact, normal conditions, therefore its threshold-decrease is expected to reflect mechanical allodynia. Since the spinal application of the two inhibitors, resulted in significant changes in the Randall-Selitto test but not in DPA, our data indicate that the two tests measure two different processes. Therefore, we can conclude that DPP4 inhibitors selectively affect hyperalgesia, but not allodynia. Our results indicate the contribution of the DPP4 to the development and maintenance of mechanical hyperalgesia in this model of neuropathy, but the underlying mechanisms are unknown. The increased expression of the enzyme was most obvious in microglia suggesting the role of this cell type in this process. The contribution of microglia to the development of allodynia in neuropathic condition is intensively studied^45–47^, but its role in the induction and maintanance of hyperalgesia and its relationship to the DPP4 requires further studies.

In this study, we demonstrated that DPP4 is expressed in the spinal cord and, similarly to that in higher brain area, its expression significantly changes during pathological conditions. Inhibitors of the enzyme do not affect acute nociceptive processing and allodynia but can selectively block glial mechanisms that contribute to the development and maintenance of hyperalgesia both in inflammatory and neuropathic conditions. This raises the possibility that DPP4 inhibitors targeting the central nervous system could be an important antihyperalgesic and anti-inflammatory component of new analgesics for the treatment of severe and persistent pain without serious side effects.

## Methods

### Animals

Nociceptive threshold measurements were carried out on male Wistar rats weighing 170–230 g and received from the breeding colony of the Semmelweis University that were used for carrageenan induced hyperalgesia model, or on animals weighing 100–160 g at the start of the partial sciatic nerve ligation experiments (Seltzer model) and obtained from Charles River Laboratories via Innovo Ltd. (Gödöllő, Hungary) or Toxi-Coop Ltd. (Budapest, Hungary). These later groups were bred and kept at the Laboratory Animal Centre of the University of Pécs. Rats were housed under similar conditions both at Semmelweis University and University of Pécs including temperature-controlled rooms with 12 h light/12 h dark cycles and standard rodent chow and tap water supplied *ad libitum*.

Experiments were performed in accordance with the European Communities Council Directive 86/609/ECC and were approved by the Committees on Animal Experiments of the Semmelweis University, Budapest (XIV-I-001/2265–4/2012) and the Medical School of the University of Pécs (BA02/2000–9/2011) Hungary.

### Drugs

Diprotin A (isoleucin-prolin-isoleucin, IPI; Sigma-Aldrich, I9759) stock solution was made up in 25% (w/v) hydroxypropyl-beta-cyclodextrin (HPβCD, Sigma-Aldrich, H107) and dilutions were made with sterile saline and administered intrathecally (i.t.) in 30 nmol/rat dose. vildagliptin (VIL) was received from Prof. Ingrid De Meester of the Laboratory of Medical Biochemistry, University of Antwerp, Wilrijk, Belgium and was administered i.t. in 3 nmol/rat dose. Naltrexone hydrochloride (NTX) was a generous gift from DuPont Pharmaceuticals (Geneva, Switzerland), and was injected subcutaneously (s.c.) in 0.5 mg/g b.w. dissolved in saline.

The MOR antagonist D-Phe-Cys-Tyr-D-Trp-Arg-Thr-Pen-Thr-NH_2_ (CTAP; Sigma-Aldrich, C6352; 200 pmol/rat), delta-opioid receptor (DOR) -antagonist H-Tyr-Tic(CH_2_NH)-Phe-Phe-OH (TIPP[Ψ]; Sigma-Aldrich, T7075; 1 nmol/rat) and kappa-opioid receptor (KOR) -antagonist 5′-guanidinonaltrindole (gNTI; Sigma-Aldrich, G3416; 10 nmol/rat) were dissolved in distilled water.

I.t. injections were delivered in 5 μl volume by a 250 μl Hamilton syringe set into a Hamilton dispenser. The 23-Ga needle was introduced at the L5–6 intervertertebral space^13,21^.

### Behavioural experiments

Nocifensive behaviours were determined by different methods to explore the inflammatory or neuropathic pain characteristics mediated by distinct anatomical and molecular mechanisms, as well as signalling pathways. The threshold decrease determined with the Randall-Selitto test applying a basically painful pressure stimulus on the rat hind paw shows mechanical hyperalgesia. The touch stimulus applied by the dynamic plantar aesthesiometry (DPA) using a blunt-end metal needle is considered to be non-painful under intact, normal conditions, therefore, its threshold decrease reflects mechanical allodynia. The changes in withdrawal latency from ice-cold water reflects cold allodynia^12^.

#### Carrageenan-induced subacute inflammation

Inflammation was induced by intraplantar injection of 100 μl l% λ-carrageenan dissolved in saline (Sigma-Aldrich, 22049) into the right hindpaw. Nociceptive threshold to pressure was determined by the Randall-Selitto method^13,21,48^ using a type 37215 Analgesimeter (Ugo Basile, Comerio, Italy). Rats were lightly restrained and an evenly increasing force was applied onto their paws inserted between the cone-shaped clamps of the apparatus. At the moment of paw withdrawal, the actual force was recorded as the nociceptive threshold. The baseline nociceptive threshold was initially determined on both hindpaws (at −5 min), then carrageenan was injected into the right hindpaw (at 0 min). The nociceptive threshold was measured again at 180 min and i.t. injection of vehicle, DPP4 inhibitors alone (IPI or VIL) or in combination with subtype specific opioid receptor antagonists (CTAP, TIPP[Ψ] or gNTI), was performed. Cardinal signs of the inflammation such as redness, swelling and heat of the paw had been obvious before the second measurement started. Nociceptive threshold readings were repeated at 185, 195, 210 and 240 minutes. Five rats were involved in each vehicle experiment, while 7–10 animals were used for drug and drug combinations.

Time-matching data sets on different ipsilateral curves were compared with two-way repeated measures ANOVA followed by Bonferroni *post hoc* test. Percentage maximum possible antihyperalgesic effects were calculated according to the following equation: MPE (%) = 100 × (ipsilateral threshold 30 min after i.t. drug application − hyperalgesic baseline)/(contralateral threshold at the same time − hyperalgesic baseline), where hyperalgesic baseline was defined as the nociceptive threshold of the inflamed hindpaw 180 min after intraplantar carrageenan injection, then comparisons were made with one-way ANOVA followed by Dunnett’s *post hoc* test^13,21^.

#### Partial sciatic nerve ligation-induced chronic neuropathic pain model (traumatic mononeuropathy)

Partial ligation of sciatic nerve results in mechanical hyperalgesia together with mechanical and cold allodynia^38,39^. Accordingly, baseline nociceptive thresholds were determined on two consecutive days using: dynamic plantar aestesiometry (DPA, mechanical allodynia), noxious cold stimulation (cold allodynia) and Randall-Selitto test (mechanical hyperalgesia). Under deep pentobarbital anesthesia (50 mg/kg i.p. Euthasol, Produlab Pharma, Raamsdonksveer, Netherlands), the sciatic nerves of rats (n = 100) were tightly ligated high in the thigh unilaterally using a braided silk suture (Mersilk 6-0, Ethicone) so that approximately 1/3–1/2 of the diameter of the nerve was trapped in the ligature. The wound was closed afterwards with 4-0 silk sutures and the animals were allowed to recover for one week.

On the 7^th^ postoperative day, nociceptive threshold measurements were repeated for each animal at short intervals and percentage hyperalgesia/allodynia values were calculated for the nerve-injured paws with the following formula: hyperalgesia/allodynia (%) = 100 × (preoperative − postoperative values)/(preoperative values). Only animals that developed a minimum of 20% decrease of threshold with each method were included in treatment groups (n = 71). Rats were arranged into groups having similar degree of hyperalgesia/allodynia and received (1) i.t. vehicle or (2) DPP4 inhibitor or (3) DPP4 inhibitor 15 min after s.c. NTX pretreatment. For i.t. vehicle and DPP4 inhibitor experiments, 8–10 animals were used in each group, while groups undergoing i.t. DPP4 inhibitor application following s.c. NTX pretreatment consisted of 5–8 rats. Nociceptive measurements from each animal were carried out 20–30 min after i.t. injection starting with DPA at 20 min, followed by the Randall-Selitto test at 25 min and finishing with noxious cold stimulation at 30 min. During dynamic plantar aesthesiometry, rats were placed into an observation chamber positioned on a metal mesh surface. The touch stimulator unit was placed under the animal’s paw and increasing upward force (10 g/s) was exerted until the rat removed its paw. Withdrawal thresholds were measured 3 times in turns for each hindpaw and the mean values were used for statistical analysis. If no withdrawal occurred, the preset maximum (50 g) was used in the evaluation. Randall-Selitto test was performed as detailed above. To measure noxious cold sensitivity, hindpaws of lightly restrained rats were immersed into a 0 °C water bath and the latency to paw withdrawal was recorded. The cut-off time was set to 180 seconds.

Withdrawal thresholds recorded before nerve ligation, then on the 7^th^ postoperative day before and after drug applications were compared with two-way repeated measures ANOVA followed by Bonferroni *post hoc* test. Percent maximum possible effects were calculated according to the following formula: MPE (%) = 100 × (withdrawal threshold after drug application − withdrawal threshold before nerve ligation)/(withdrawal threshold before drug application − withdrawal threshold before nerve ligation), then comparisons were made with one-way ANOVA followed by Dunnett’s *post hoc* test.

### RNA isolation and real-time PCR analyses

Carrageenan was injected into both hindpaws of 6 rats and partial ligation of both sciatic nerves was performed in 9 animals. Survival time was 3 hours and 7 days, respectively. Development of inflammatory or neuropathic hyperalgesia was confirmed by the Randall-Selitto test, and then rats were sacrificed by decapitation, with a further 6 animals used as controls. L4–L6 spinal segments were removed and frozen on dry ice. RNA was isolated using the RNeasy Lipid Tissue Mini Kit (QIAGEN) from spinal cord samples according to the manufacturer’s instructions. The purity and concentration of the RNA were analyzed using a SmartSpec Plus spectrophotometer (Bio-Rad, UK). Reverse transcription was performed with 1 μg of RNA to convert the total RNA to cDNA using the High-Capacity cDNA Reverse Transcription Kit (Applied Biosystems by Life Technologies). Concentration of the generated cDNA was determined using the Qubit 2.0 Fluorometer with the Qubit ssDNA Assay Kit (Life Technologies). Expression of DPP4 mRNA was measured by real-time quantitative TaqMan RT-PCR reaction with a ViiA 7 Real-Time PCR System (Life Technologies), using commercially available TaqMan probe (Rn00562910_n1) on 10-ng cDNA template in duplicates. Glyceraldehyde-3-phosphate dehydrogenase (Rn99999916_s1) was used as a housekeeping gene, and its expression did not vary between the experimental groups.

For statistical analyses, qPCR data were expressed as relative quantification values (RQ; mean ± SEM) and compared between groups by one-way ANOVA.

### *In situ* hybridisation

The 1632–2051 bp long fragment of the rat DPP4 cDNA (gene bank accession #NM_012789) was purchased from Blue Heron Biotechnology Inc. (Bothell, WA, USA), subcloned into pBC KS + **(**Addgene, Cambridge, MA, USA) vector, and verified by sequencing. *In situ* hybridisation (ISH) was performed as described earlier^49^. Riboprobes in sense and antisense directions were prepared by *in vitro* transcription (MAXIscriptKit, Life Technologies, Carlsbad, CA, USA) and labelled using [^35^S]UTP-(Per-Form Hungaria Kft, Budapest, Hungary). Carrageenan was injected into both hindpaws of 3 rats and partial ligation of both sciatic nerves was performed in 3 animals. Survival time was 3 hours and 7 days, respectively. Development of inflammatory or neuropathic hyperalgesia was confirmed by the Randall-Selitto test, and then rats were sacrificed by decapitation, with a further 3 animals used as controls. The spinal dorsal horn of L4-L6 segments were removed and frozen on dry ice. Serial coronal sections were cut in a cryostat and mounted onto positively charged Superfrost Plus slides (Life Technologies). Slides were hybridized overnight in humid chambers at 55 °C with 10^6^ cpm/slide of the radioactively labelled probes, washed and dehydrated. Slides were dipped into NTB nuclear track emulsion (Carestream Health Deutschland GmbH, Stuttgart, Germany) for 4 weeks. Emulsion-coated slides were developed using Kodak Dektol developer and Fixer (Sigma-Aldrich Kft, Budapest, Hungary). Sections were counter-stained with 0.5% Giemsa solution (Sigma), air dried and coverslipped using Depex mounting medium. Dark-field images of three samples per animal were captured by a BX51 Olympus microscope (Olympus Corporation, Hamburg, Germany) attached to a QICAM (Qimaging, Surrey, BC, Canada) camera. The grain density indicating the level of the DPP4 mRNA expression was calculated as the area percent occupied by the silver grains within a given region of interest (ROI; 100 × 100 pixel^2^) using the Image J 1.32j program. In each section, 3 ROIs from the background (area outside of the tissue) and 5 ROIs from the dorsal horn were measured and averaged. Then the background was subtracted from the value that was obtained from the tissue. The sense and antisense signals were compared using Student’s t-tests with SigmaStat 3.5program (Systat Software, Inc. San Jose, CA, USA). One way ANOVA was used for comparing the antisense *in situ* hybridization signals among the groups.

### Western blotting

Five rats with unilateral carrageenan-induced hindpaw inflammation, 9 rats undergoing unilateral partial nerve ligation one week earlier with further 4 naïve rats were tested with the Randall-Selitto method as it was described above. After confirming the obvious decrease of nociceptive thresholds, animals were sacrificed by decapitation, and the spinal dorsal horn of L4–L6 segments were removed and snap-frozen on dry ice. Samples were homogenized in TNE buffer containing 0.5% Triton X-100 (Sigma), 5 mM NaF, 100 μM Na_3_VO_4_ and a cocktail of protease inhibitors (CompleteTM, Roche) and briefly sonicated. Cell debris and nuclei were pelleted by centrifugation (800 g, 30 min at 4 °C). Protein concentrations were determined by Bradford’s colorimetric method^50^. Samples were diluted to a final protein concentration of 2 μg/μl, denatured in 5x Laemmli buffer, and analysed by SDS-PAGE on a 10% resolving gel. After transferring onto Immobilon-FL polyvinylidene difluoride membranes (Millipore), membrane-bound protein samples were blocked in 3% BSA and 0.5% Tween-20 diluted in TBS for 1.5 h, and subsequently exposed to the goat DPP4 primary antibody **(**Table 1.) overnight at 4 °C. Signal detection was achieved by using a HRP-conjugated donkey anti-goat secondary antibody (Jackson; 1:10,000). Densitometric data were normalized to β-actin and for quantification three blots per treatment were used. Image acquisition and analysis were performed on a Bio-Rad XRS + imaging platform.

### Immunofluorescent labelling

#### Antibodies

Polyclonal goat DPP4 antibody was raised against the synthetic peptide C-PPHFDKSKKYP representing the internal region of DPP4 according to NP_001926.2 and labelled one band at approx. 110 kDa in rat lung lysate in Western blot experiments (for details see the supplier’s datasheet). In our Western blot experiments rat lung and pancreas lysates were used as positive controls (Fig. 1c). Monoclonal mouse DPP4 antibody was produced against the full length rat CD26 protein. To test the specificity of the two DPP4 antibodies double immunofluorescent staining was carried out^51^. Both antibodies labelled the same profiles in neurons, astrocytes and microglia (Fig. 2).

Mouse anti-neuron-specific nuclear protein (NeuN) was used for labelling neuronal somata^52^ together with NeuroTrace 435/455 blue-fluorescent Nissl stain^53^ (Thermo Fischer Scientific - Invitrogen, Cat#: N-21479; 1:200). Dendrites were identified with monoclonal mouse antibody produced in mice against microtubule-associated protein 2 (MAP2)^54,55^. Antibody stained one single lane at 280 kDa in Western blot experiment using rat brain extract. Ionized calcium-binding adaptor molecule-1 (IBA1) was used as specific microglia/macrophage marker and anti-IBA1 antibody was isolated from the serum of rabbits immunized with a synthetic peptide corresponding to C-terminus of IBA1. According to the provider antibody stains one single lane in Western blot experiments at around 17 kDa. Monoclonal mouse antibody against glial fibrillary acidic protein (GFAP) was used for specific labelling of astrocytes. Guinea pig antibody against vesicular glutamate transporter 2 (VGLUT2) was used to identify excitatory axon terminals. The antibody is raised against a 18 amino acid long sequence of the rat protein and Western blot analysis on rat brain lysate showed 52 kDa lane as it is seen on the provider’s datasheet related to Lot#: NG1866937. This VGLUT2 antibody is heavily used and well characterized^56^. Inhibitory boutons were labelled with polyclonal antibodies against vesicular GABA transporter (VGAT) raised in rabbits immunized by the synthetic peptide AEPPVEGDIHYQR (amino acids 75–87 in rat VGAT) coupled to key-hole limpet hemocyanin via an added N- terminal cysteine. Based on the supplier’s data sheet this antibody is knock-out verified. Synaptophysin (SYN) was used as a synaptic marker and monoclonal mouse antibody was raised against synthetic peptide corresponding to a region near to the C-terminal end of the full peptide. SYN antibody validation by immunohistochemistry and Western blot is found in the Human Protein Atlas (http://www.proteinatlas.org). Calcitonin gene-related peptide (CGRP) antibody raised in guinea pig and used for labelling peptidergic unmyelinated primary afferents recognizes identical structures to those detected by well characterized rabbit and goat antibodies against rat α-CGRP^57^. For detailed specifications of antibodies see Table 2.

**Table 2:** Specifications of primary antibodies used for immunohistochemistry.

Antibody	Species	Dilution	Source	Catalog No.
CGRP	guinea pig	1:5000	Bachem-Peninsula Laboratories	T-5027
DPP4	goat	1:500	Fischer Scientific-Novus Biological	NB100-61658
DPP4	mouse	1:500	Abcam	ab119346
GFAP	mouse	1:100	Leica Biosystems-Novocastra	NCL-GFAP-GA5
IBA1	rabbit	1:500	Wako Pure Chemical Industries Ltd	019–19741
MAP2	mouse	1:500	Sigma-Aldrich	M9942
NeuN	mouse	1:1000	Merck-Millipore-Chemicon	MAB377
VGAT	rabbit	1:1000	Synaptic Systems	131 002
VGLUT2	guinea pig	1:5000	Merck-Millipore-Chemicon	AB2251
Synaptophysin	mouse	1:1000	Leica Biosystems-Novocastra	NCL-SYNAP-299

Secondary antibodies were all raised in donkey: Alexa Fluor (AF) -488 labelled anti-goat, AF-555 conjugated anti-mouse, AF-555 labelled anti-rabbit (all from Thermo Fischer Scientific-Invitrogen-Molecular Probes; 1:500) and Rhodamine Red X-anti guinea pig, Cyanine 5-anti mouse, Cyanine 5-anti rabbit (all from Jackson ImmunoResearch; 1:100).

#### General immunofluorescent staining protocol

Transcardial perfusion of deeply anesthetized (75 mg ketamine and 7.5 mg xylazine; i.m.) rats (5 naive, 5 unilateral carrageenan treated, and 5 unilateral nerve ligated as described above) was initiated with 4% (para)formaldehyde and completed with 4% (para)formaldehyde containing 10% sucrose. L4-L5 spinal segments were removed and immersed overnight into 20% sucrose dissolved in PBS. Segments were frozen with liquid nitrogen and then 50 µm thick sections were cut on a Vibratome. Endogenous peroxidase activity was blocked for 30 minutes with 1% hydrogen peroxide diluted in phosphate buffer (PB), then sections were transferred into phosphate buffered saline (PBS) with 5% normal horse serum (NHS). After the blocking procedure they were incubated overnight in the DPP4 antibodies then reacted with Alexa Fluor 488 labelled species specific secondary antibodies. Sections were incubated for 72 hours in mixtures of the other primary antibodies and were reacted overnight with fluorescently labelled species specific secondary antibodies. All the primary and secondary antibodies were dissolved in PBS. In some sets of experiments, sections were immersed into Neurotrace fluorescent Nissl dye (Invitrogen-Molecular Probes; 1:200 in 0.1 M phosphate buffer) for half an hour before mounting. After rinsing, sections were mounted in Vectashield (Vector Laboratories) and scanned on a confocal laser scanning system (Zeiss, LSM780).

#### Densitometry of DPP4 immunostaining

L4 and L5 spinal cord segments taken from 5 control, 5 carrageenan-treated and 5 nerve-ligated rats were used for quantitative analysis. Three to six spinal cord sections were taken from each segment on the basis of the gray matter shape and 5 to 8 confocal optical sections were scanned from each section. DPP4 staining was not viewed prior to selecting any sections, ROIs or cell profiles. The quantitative analysis was carried out by an independent observer, who was blind to the experimental conditions and was not involved in the scanning either.

To determine DPP4 immunoreactivity and its alteration under different circumstances fields containing the whole dorsal horn were scanned through a 20x lens of the confocal microscope to produce z-stacks with z separation of 1 µm. The scanning parameters were selected and optimized in sections from control animals and were used further in sections of treated rats. Single optical sections containing black and white images were selected from each z-series and analysed using the Image J program (Rasband WS, Image J, NIH, Bethesda, Maryland). The medial two third of the dorsal horn containing the first four laminae (the area receiving inputs from the sciatic nerve) was drawn in each section and used as region of interest (ROI). The threshold was adjusted and the density of the immunostaining was calculated as the area percentage occupied by the immunostained dots within a given ROI. Data were compared among groups by one-way ANOVA.

To analyse the density of the DPP4 immunoreactivity in individual cell types, non-overlapping fields of 135 µm × 135 µm within the medial two third of the spinal dorsal horn were scanned through a 63x oil immersion lens to generate z-stacks with a z-separation of 0.5 µm. The same optimized parameters were used for all types of sections. IBA1-, GFAP- and DPP4-immunolabellings (for glial cells) or DPP4 immunolabelling with fluorescent Nissl staining (neuronal cell bodies) were imaged in different colour channels sequentially. Outlines of microglia and astrocytes were determined automatically by using the AutoThreshold plugin of ImageJ in IBA1 and GFAP image channels, respectively. To exclude non-specific labelling of glial cells and nuclei by the fluorescent Nissl dye, contours of randomly selected neurons (8 neuron/field) were drawn manually. Then integrated density values from DPP4 image channel were measured in previously delineated glial or neuronal profiles to detect changes in protein expression due to different treatments. The density values were subjected to statistical analysis.

### Statistical analysis

Statistical methods used are detailed at each experiment individually. Analyses were made with SigmaStat 3.5program (Systat Software, Inc. San Jose, CA, USA) and curves/bar graphs were created with the GraphPad Prism 5.0 software (GraphPad Software Inc., La Jolla, CA, USA). In general, data were represented as mean ± SEM when the population was normally distributed or as median with 25% and 75%, otherwise. In both cases, p < 0.05 was considered as statistically significant.

### Data Availability

The datasets generated and analysed during the current study are available from the corresponding author on reasonable request.

## Electronic supplementary material

Supplementary Figure S1
