# Supplementary material for: Glial cell type-specific changes in spinal dipeptidyl peptidase 4 expression and effects of its inhibitors in inflammatory and neuropatic pain
Source: Sci Rep. 2018 Feb 22;8:3490. doi: 10.1038/s41598-018-21799-8 (PMC5823904; doi:10.1038/s41598-018-21799-8)
Supplement: Supplementary file 1 — Supplementary Figure S1 [file 41598_2018_21799_MOESM1_ESM.pdf]

Glial cell type-specific changes in spinal dipeptidyl peptidase 4 expression and effects of its inhibitors in inflammatory and neuropatic pain

Authors: Kornél Király, Márk Kozsurek, Erika Lukácsi, Benjamin Barta, Alán Alpár, Tamás Balázsa, Csaba Fekete, Szabon Judit, Zsuzsanna Helyes, Kata Bölcskei, Valéria Tékus, Zsuzsanna E. Tóth, Károly Pap, Gábor Gerber, Zita Puskár

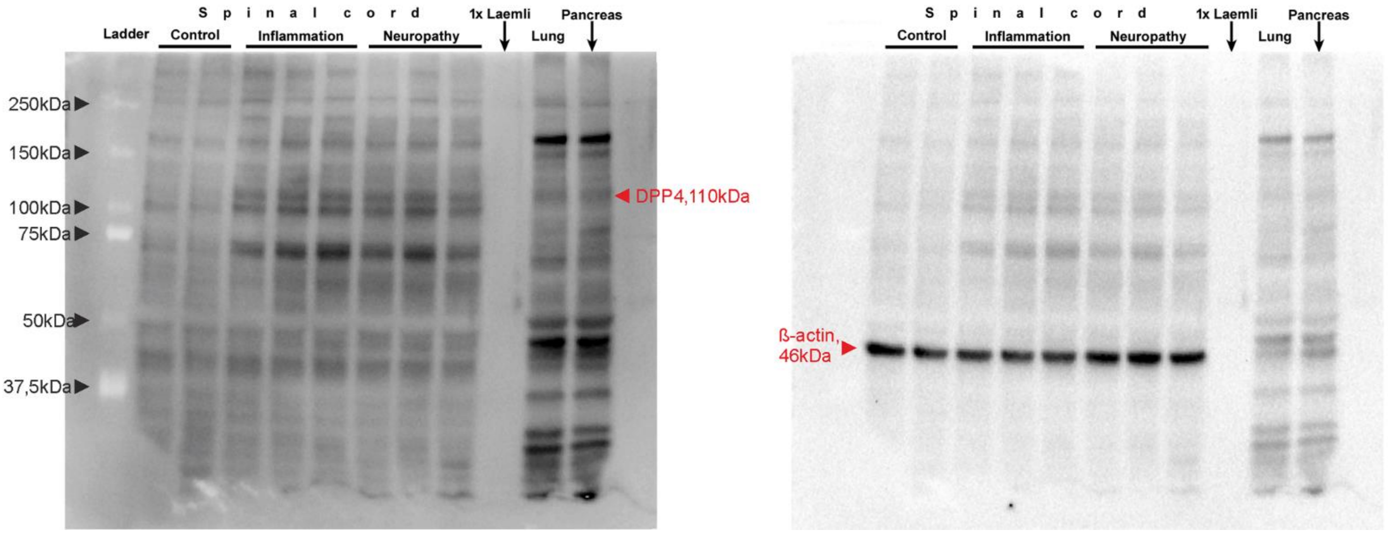

Supplementary Figure S1. The complete membrane of whole lane Western blots run on a 6% SDS gel and processed for DPP4 immunohistochemistry. Protein free 1xLaemmli solution and lung/pancreas tissues were used as negative and positive controls, respectively. The blotting membrane was incubated first with DPP4 antibody then after stripping with anti-β actin as loading control.
